# Designing a food supply chain strategy during COVID-19 pandemic using an integrated Agent-Based Modelling and Robust Optimization^☆^

**DOI:** 10.1016/j.heliyon.2021.e08448

**Published:** 2021-11-19

**Authors:** Audi Luqmanul Hakim Achmad, Diah Chaerani, Tomy Perdana

**Affiliations:** a Master of Mathematics Study Program, Faculty of Mathematics and Natural Sciences, Universitas Padjadjaran, Indonesia; b Department of Mathematics, Faculty of Mathematics and Natural Sciences, Universitas Padjadjaran, Indonesia; c Department of Agro Socio-Economics, Faculty of Agriculture, Universitas Padjadjaran, Indonesia

**Keywords:** Food supply chain, COVID-19, Robust Optimization, Agent-Based Modelling, Uncertainty, Simulation

## Abstract

Coronavirus disease (COVID-19) has spread for over a year and affected many aspects, including the food supply chain. One of the ways COVID-19 has impacted the food supply chain is the food production capacity reduction. It is necessary to develop the optimum food supply chain strategy by determining the optimum food hub location and food network to maintain food security which robust against disruptions and uncertainties. In this study, Robust Optimization (RO) is applied to handle the uncertainties. Nevertheless, the actual uncertain data might be hard to be collected or even unavailable at the moment. Therefore, an innovative framework is proposed to integrate RO with Agent-Based Modelling (ABM). ABM is used to simulate the upstream actor of the food supply chain and predict the uncertain food production capacity, which RO later handles. Particularly, this study focused on rice supply chain. The result shows that the framework is able to handle the uncertain rice supply chain problem, in which the actual uncertain data might be unavailable, and give the robust optimum food hub location and food network. The food hub location and food network are obtained by solving the Robust Counterpart (RC) model with respect to the uncertainty set obtained from the ABM simulation result.

## 1 Introduction

According to WHO (2020a), COVID-19 was first emerged in Wuhan, China, on 31 December 2019. It was first identified in Indonesia on 02 March 2020 (Suryahadi et al., 2020). Several attempts have been made to control the spread of COVID-19, including travel restrictions (Min et al., 2020; Pulighe and Lupia, 2020; Singh et al., 2020). Border restrictions between countries are also applied temporarily (Chen et al., 2020; Lawson-Lartego and Cohen, 2020; Zhao et al., 2020). These restrictions affected many aspects, including the food supply chain.

One of the impacts on food supply chain is the reduction of food production rate in several commodities (Mahajan and Tomar, 2020; Udmale et al., 2020). Everyone is at risk to become infected by the virus, including the workers along the food supply chain. In this case, any infected workers have to isolate themselves for a couple of days and caused the labour force in food supply chain becomes decreased, which results in the reduction of food production rate (Abiral and Atalan-Helicke, 2020; Kodish et al., 2019; Ma et al., 2020). The reduction of food production rate leads to the reduction of food production capacity (Butu et al., 2020; O'Hara and Toussaint, 2021). Agricultural production in Southeast Asia is estimated to be decreased by 3.11% (around 17.03 million tons) in the first quarter of 2020 because of the mobility restriction (Gregorioa and Ancog, 2020). In Indonesia, agricultural production is decreased by 3.28% (around 2000 tons) in the first quarter of 2020. Furthermore, another impact is the decreased speed of food movement among the segments in food supply chain (Butu et al., 2020; O'Hara and Toussaint, 2021; Luckstead et al., 2020). The reduction of food production capacity and food movement speed affect food availability (Sukhwani et al., 2020). Therefore, developing the optimum food supply chain strategy is necessary to maintain food availability and guarantee food security.

Many studies have been discussed pandemic impacts in food supply chain. COVID-19 impacts in food production and food consumption patterns are discussed by Galanakis C.M. (2020), Hobbs (2020), and Mukhamedjanova (2020). Meanwhile, HIV/AIDS impacts on food production in Nigeria is discussed by Chucks (2008). Another discussion is also given by Mohan et al. (2009) and de Krom and Mol (2010), which studied the impact of influenza in the poultry food supply chain and food consumption patterns.

However, to date, the number of studies that proposed a food supply chain strategy involving pandemic conditions is still limited. Food reliefs distribution strategy during a pandemic is given by Ekici et al. (2014). They proposed the food relief distribution during the influenza pandemic to minimize the total cost needed. Meanwhile, food supply chain strategy is given by Nagurney (2021), which focused on maximizing the profit of food supply chain during COVID-19 condition. Both of the studies have not considering any uncertainty. The discussion of food supply chain strategy during a pandemic, which also considers uncertainty, is discussed by Perdana et al. (2020). They studied three scenarios of food supply chain during COVID-19, which considered food consumption, food production, and food distribution cost uncertainty. Nevertheless, one gap of the study is the use of historical data (food production and food consumption data) of the last ten years for the uncertainties instead of using the actual data during COVID-19 since it is unavailable at the time. The actual production and consumption data are difficult to be obtained during a disaster, including pandemic (Kartez and Lindell, 2007). To fill the gap of our previous study, a possible framework is proposed to be applied as an alternative to obtain the robust optimum food supply chain strategy involving normal and pandemic situations by using the data provided by simulation as the uncertainties when the actual data is unavailable.

This paper proposes an integrated framework consisting of the Agent-Based Modelling (ABM) and Robust Optimization (RO) approach to solve food supply chain problems under uncertainties involving normal and pandemic conditions. Particularly, this study focused on the rice supply chain since rice is the typical staple food in Indonesia (McCulloch and Timmer, 2008; Supriatna et al., 2019). The novelty of this study is the proposed approach to integrate ABM and RO in solving the food supply chain problems by designing a food supply chain strategy through the determination of food hub location and food network with data availability issues. Based on a literature search in the Scopus database, no article has been found in regards to integrate ABM and RO in solving food supply chain problems. Nevertheless, there are a few articles that applied the integration of ABM in RO in two different subjects, namely, energy management and oil supply chain.

Although this study focused on the rice supply chain problem, the proposed framework is applicable to any food supply chain problem in which the system could be described as a collection of components with their respective behaviour. From the broader perspective, this integrated simulation and optimization framework could be developed and implemented to optimize the future state of a system, in which the simulation is used to generate outcomes through several scenarios and provide the input parameters needed by the optimization model (Beraldi et al., 2010).

This study uses an ABM to simulate the rice production system during a pandemic and give the prediction data of rice production capacity. Based on the predicted rice production capacity from the ABM simulation, two scenarios are generated to be further analyzed and handled with RO. RO is applied to handle the dynamic rice production from the simulation result. The first scenario uses the whole data from all simulation repetitions. Meanwhile, the second scenario takes the highest 15% of rice production capacity.

This paper is organized as follows: Section 2 discusses several articles related to this study. Section 3 discusses the methods used in this framework, which consists of the ABM model and the uncertain optimization model used in RO, followed by the framework integration of ABM and RO. Section 4 gives the results and discussion of the integrated ABM and RO in the rice supply chain through a case study. Section 5 gives the conclusion of this study.

## 2 Literature review

RO is one of the methods in optimization to handle uncertainties involved in the optimization model (Gorissen et al., 2015; Yanıkoğlu et al., 2019). One of the advantages of RO over stochastic optimization is that it does not require any prior information about the probability density function of the uncertainty, which is often difficult to be obtained (Bohle et al., 2010). Rather, RO will handle the uncertain data by assuming all uncertainties are gathered in a single convex hull called uncertainty set. Regarding the food supply chain, RO plays a big role since various uncertainties have become the challenges in both demand and supply-side of the chain (Wan et al., 2016). One example of the studies in food supply chain involving uncertainties is given by Behzadi et al. (2013). They propose a model to optimize cold food supply chain logistics involving demand uncertainty to maximize food safety with the minimum costs possible. Another study is also given by Maiyar and Thakkar (2020), which discussed intermodal transport optimization in food grain supply chain under uncertain procurement. Our study used RO to optimize the rice supply network, maximizing demand fulfillment and minimizing operational cost with uncertain rice production capacity affected by COVID-19. Since the actual data of rice production are difficult to be obtained, an ABM is developed to simulate and provide the predicted rice production capacity affected by COVID-19.

ABM is a simulation method that has the advantage to simulate a system based on the actors/agents involved (Macal, 2016). ABM specifies all actors in the system along with their behaviours and how they interact with other agents and their environment (Chen et al., 2013; Zhang et al., 2019). In the domain of food supply chain, the benefits of ABM are widely used to explore the behaviour of numerous actors along the chain, explaining their behaviour and its impact from numerous perspectives (Utomo et al., 2018). One study of assessing the impacts of entrepreneurship capabilities in agri-food markets is given by Ross and Westgren (2009). They develop the agents with and without entrepreneurial capabilities through their behaviours in operating their business. Through the simulation, they are able to point out several key entrepreneurial capabilities to enhance the firm performance. Whereas, Vojnovic et al. (2020) studied the food shopping behaviour via ABM simulation. They observed factors affecting the food shopping behaviour by constructing the agents' behaviour based on survey data of food access. The study gives an insight of how those factors might affect food consumption.

To justify the novelty of this study, a literature search of ABM and RO in the food supply chain is conducted. The topic of this study is the integration of ABM and RO in the food supply chain. Five criteria are applied to obtain the preferred articles related to this topic; (1) peer-reviewed articles, (2) articles written in English, (3) published by journals, (4) using ABM and RO as their methods and (5) applied the methods to solve food supply chain problems. This literature search is performed using the Scopus database since it is the largest database of peer-reviewed articles (Gusenbauer, 2019). To satisfy all criteria, the keywords used in this literature search are:

(“Robust Optimization” OR “Robust Optimisation”) AND “Agent-Based Model*” AND “Food Supply *” AND (LIMIT-TO(DOCTYPE,”ar”)) AND (LIMIT-TO(LANGUAGE,”English”)) AND (LIMIT-TO(SRCTYPE,”j”))

By using the given keywords, no article was found. This result indicates that there has not been any study integrating ABM and RO in food supply chain problems at the moment. Therefore, this becomes the novelty of this study in solving food supply chain problems.

To get insight into the current works related to this topic, another literature search with the generalized topic is performed. The generalized topic is ABM and RO with no specified subject. Four criteria are applied to obtain the new generalized topic; (1) peer-reviewed articles, (2) articles written in English, (3) published by journals and (4) using ABM and RO as their methods. To satisfy the four criteria, keywords used in this literature search are:

(“Robust Optimization” OR “Robust Optimisation”) AND “Agent-Based Model*” AND (LIMIT-TO(DOCTYPE, “ar”)) AND (LIMIT-TO(LANGUAGE,”English”)) AND (LIMIT-TO(SRCTYPE,”j”))

By using the Scopus database, five articles are obtained. Among the five articles obtained, two of them did not relate to RO. Thus, there are three articles relevant to this topic. The three articles apply ABM and RO in two different subjects: oil supply chain and energy management. The discussion of ABM and RO integration to solve oil supply chain problems is given by Hu et al. (2012). They used ABM to simulate the oil refinery process and market business. Oil refinery and customer agents are used in their simulation model, in which the goal is determined from the oil refinery management perspective, e.g., maximize profit and maximize oil product quality. Therefore, they built the oil refinery agents with the ability to optimize their decision during the simulation by using the Robust Counterpart (RC) model. The uncertainties in the RC model are assumed to be known in the form of interval. Another similar framework is also proposed by Kuznetsova et al., which studied the integration of ABM and RO in energy management problems (Kuznetsova et al., 2015, Kuznetsova et al., 2014). They built an ABM simulation model, which consists of the wind power plant, train station, and local community as the agentset with the ability to optimize their decision during the simulation based on the RC model. The novelty of this framework is the approach to generate the interval uncertainties instead of assumed to be known. They used Neural Network (NN) trained by Non-dominated Sorting Genetic Algorithm-II (NSGA-II) to estimate the interval uncertainties. Using the estimated interval uncertainties, they run the simulation model and let each agent optimize their own decision with respect to the RC model. Both of the studies have a similar framework, in which they used the RC model from RO to be implemented in ABM simulation.

In this study, a different framework of ABM and RO integration is proposed. The proposed framework in this study is using ABM simulation as a tool to generate the uncertain data and use it to obtain the uncertainty set. Then, the uncertainty set is used in RO to solve the RC model with respect to the obtained uncertainty set. Particularly, ABM is used in this study to simulate the pandemic spread and its impact on rice production capacity. The rice production capacity data from the simulation are then processed with RO to obtain the uncertainty set. Then, the RC model is solved based on the uncertainty set obtained from the simulation.

## 3 Methods

To solve uncertain food supply chain problems with limited production data available, a framework that integrates ABM and RO is proposed. This section gives the brief concept of ABM and RO used in this study, followed by the framework of integration between ABM and RO. ABM is used to simulate the upstream actor, particularly rice milling unit workers, in processing the rice grain into the rice. The output of ABM is the rice production data, which later be handled in RO to obtain the robust optimum supply chain design through food hub location and food network.

### 3.1 Agent-Based Modelling

Agent-Based Modelling (ABM) is a simulation method to analyze a complex and dynamic system using agents as the representation of components involved in the system (Zhang et al., 2019). Agents are constructed to mimic the behaviour and its interaction with other agents (Mashhadi Ali et al., 2017). In general, there are three main elements of ABM: (1) agentsets (and its characteristics), (2) interactions among the agentsets, and (3) behaviour between agents and environment (Zhang et al., 2019; Macal and North, 2010; Wall, 2016). Therefore, it is necessary to identify the three elements of the system which are going to be simulated.

In this framework, ABM is developed based on the model which has been proposed by Mahdizadeh Gharakhanlou and Hooshangi (2020). The ABM model used in this framework is given by describing the three main elements of ABM as discussed in the following subsubsection.

#### 3.1.1 Agentsets

To simulate the spread of the disease and its impact on food production capacity, three agentsets are used in this model: (1) human, (2) houses, and (3) Rice Milling Units (RMU). Based on the types of its movement, the three agents can be classified into two types: static agents and dynamic agents. In this model, human is the only dynamic agent. Meanwhile, houses and RMU are classified as static agents. Attributes correspond to each of the agentset is given in Table 1.

**Table 1:** Attributes of each agentset.

Agentset	Static/Dynamic	Attribute
House	Static	House ID
		Spatial location
RMU	Static	RMU ID
		Spatial location
		Quantity of food produced
Human	Dynamic	Human ID
		Hometown
		House location
		Current location
		Job
		Job location
		Current activity
		Activity duration
		Health status
		Recovery time
		Quarantine status
		Infecting probability
		Death probability

Each human agent has an ID to distinguish a human from others and remains unchanged during the simulation. Therefore, ID is a static attribute. Other static attributes which remain unchanged during the simulation are hometown, house location, and job. House location represents where the human agent lives, and hometown represents the city where the house is located. In this model, five types of jobs are considered: (1) Rice Milling (RM) workers, (2) civil servants, (3) private employees, (4) students, and (5) freelancer. Based on their own job, each human has their own activities observed in current activity. The duration of activity which has been done by each agent is recorded in activity duration. They also have their own location to do their activities (schools for students, RMU for RM workers, offices for civil servants and private employees). Meanwhile, freelancers are assumed not to have a particular location to do their activities.

**Figure 1:**
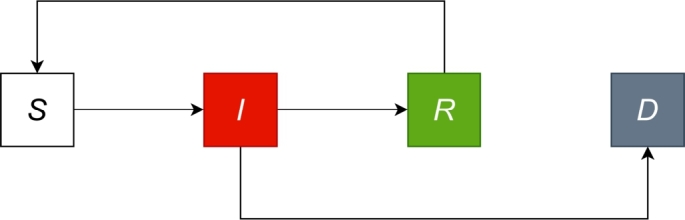
Health status transition of human agents.

Furthermore, each human agent also has a health status. In this model, we consider four types of health status as adopted from the epidemiological model: Susceptible, Infected, Recovered, and Dead (SIRD). The possible health status transition of human agents along the simulation is given in Fig. 1.

Susceptible individuals are humans who have the risk of becoming infected. In this model, we assume that all humans are susceptible with a certain probability of becoming infected when they interact with or near the infected humans. This probability is set based on Indonesia's Infection Rate (IR), as given by Rahmandad et al. (2021). The infected status considered in this model is divided into two categories: (1) detected and (2) undetected. The undetected infected human is an infected human who did not develop any symptom, also known as asymptomatic. The undetected infected individuals are doing their activities as usual. They have a probability of becoming detected. This probability is set based on the measured ratio between detected and undetected cases for Indonesia, as given by Ndii et al. (2020). Meanwhile, the detected infected individuals have to undergo quarantine as long as they are still being infected. The detected and undetected infected individuals have their own recovery time, which is randomly set based on an interval. The interval used in this model is set based on the report given by WHO (2020b). After passing the recovery time, the infected individuals have two possibilities: (1) recovered or (2) dead, with a certain probability. The death probability of infected humans is set based on Infection Fatality Rate (IFR) measured for Indonesia given by Rahmandad et al. (2021). The recovered individuals can become susceptible with a certain probability, represented as “Recovered to Susceptibility Probability”. The value of several input parameters used in this model is given in Table 2. More details on disease spread among human agents are covered in Subsubsection 3.1.2, which discusses the interactions among the agentset.

**Table 2:** Several input parameters used in the model.

Input parameter	Value	Reference
IR	0.337	(Rahmandad et al., 2021)
Detected Cases Ratio	0.51	(Ndii et al., 2020)
Minimum Recovery Time	14 days	(WHO, 2020b)
Maximum Recovery Time	42 days	(WHO, 2020b)
IFR	0.0188	(Rahmandad et al., 2021)
Recovered to Susceptible Probability	0.90	Authors' estimation

House agents is a simple static agent with ID to distinct one house and others. This agent also has spatial location attribute as coordinate and city in which the agent is located. House agent is used to generating the initial population of human. Meanwhile, RMU agents have the quantity of food produced as their attribute to collect the total of food produced in RMU as RM workers work in the RMU location. More of the details about the relation of human and RMU agents are described in the following subsection.

#### 3.1.2 Interactions among the agentsets

In this model, the primary interaction occurs among the human agents. The interaction among the human agents is disease spread. When any susceptible human is near or passing any infected individual, they become infected with a certain probability. The infected individuals have their own recovery time. Undetected infected individuals are doing their activities as usual during their recovery time. Meanwhile, detected infected individuals are going to be quarantined until they finished their recovery time. After they passed recovery time, they both have the same possibilities: recovered or dead, with a certain probability. The recovered individuals can be returned to susceptible with a certain probability.

**Figure 2:**
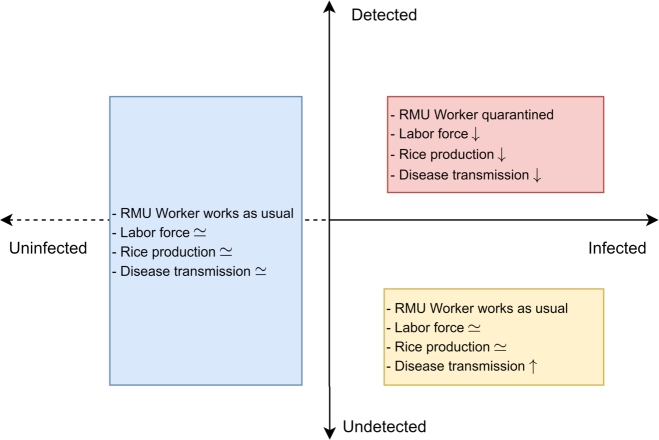
COVID-19 impacts on RM workers and rice production.

Moreover, there is also another interaction in our model, which occurred between humans and RMU agents. Mainly, the interaction occurs between human agents who worked as RM workers and RMU agents. RM workers are working in RMU. When they are working at their unit, they will process the rice grains to produce rice. The produced rice is then stored in RMU, which is observed through the quantity of food produced as an attribute of RMU agents. When the RM workers are uninfected, they are working, as usual, to process rice grain into the rice as presented in Fig. 2. When RM workers are infected and detected, they become quarantined. The quarantined RM workers lead to a decreased labour force in RMU, which eventually reduce rice production. Meanwhile, when the RM workers are infected undetected, they are still doing their activities, processing the rice grain into the rice. Although rice production is not disrupted, the disease transmission could be increased.

#### 3.1.3 Interactions between agents and environment

This simulation is run with two conditions applied consecutively: (1) during the normal condition (COVID-19 has not emerged) and (2) during the COVID-19 condition. The simulation begins with the normal condition for one year. Then, the COVID-19 condition is set to emerge in the simulation for the following year. There are two possible scenarios when the COVID-19 condition is applied: (1) with travel restriction applied and (2) without travel restriction. This travel restriction is applied within the region/city level. When any city applied travel restrictions, the activities of human agents are restricted. Under travel restrictions, 75% of the work time of civil servants and private employees are taken from home, and only 25% of them are taken from their respective offices. Students are forced to study from their homes during this travel restriction. Meanwhile, any essential sector is fully operated. In this case, RM workers are still allowed to work from their RMU to produce rice as usual. The decision whether a city is applied travel restriction or not is taken based on three criteria: (1) if the death rate of any city is higher than the average whole death rate among the cities, (2) if the recovery rate of any city is lower than the average recovery rate among the cities, and (3) if the number of active cases of any city is higher than the average of active cases among the cities. To achieve this, all human agents are continuously gathering the travel restriction status of its environment in their current city. If travel restriction is applied in any city, the human agents are adapting to the situation and behave as described.

#### 3.1.4 Model validation

There is no definitive approach to validate the simulation model (Law and McComas, 1991). To verify input parameters used in the ABM simulation, sensitivity analyses are done. One example of sensitivity analysis is for the “Recovered to Susceptibile Probability” estimation, or re-susceptible probability. We tested several numbers of re-susceptible probability as given in Table 3 and observe the impact on the number of detected new cases. We tested out 100 simulation repetitions for each of the probability options. Based on the analysis, it is observed that a phenomenon of multiple waves for the new cases occurs among 87 simulation runs out of 100 in the case of 0.9 re-susceptible probability. High re-susceptible probability indicates the high risk of people being re-infected, which sustains the total number of infected people in a relatively long period. Hence, the uninfected individuals are at high risk to become infected, resulting in new waves of new cases once they are infected. The multiple waves observed mainly consist of only two waves, with a rare occurrence of three waves and above (since the simulation under COVID-19 is run within a year). When the re-susceptible probability is lower, the whole infected case is also sustained in a shorter period, lowering the risk of uninfected individuals becoming infected. In other words, if there are not enough infected individuals to spread the disease, then new waves of infected cases will not occur. Therefore, we choose the value of 0.9 as re-susceptible probability for the simulation since multiple waves of COVID-19 are occurred in reality.

**Table 3:** Sensitivity analysis of “Recovered to Susceptible Probability” input parameter.

Re-susceptible input parameter	Number of simulations	
	Multiple waves	Single wave
0.9	87	13
0.8	68	32
0.7	65	35
0.6	52	48

Another way to validate a simulation model could be done by comparing the measures from the simulation model with the measures of the existing system Law and McComas (1991). The simulation model is said to be “valid” if the measures from the simulation model are “close” to the measures of the existing system. If no actual measures are found, one can validate the simulation model by assessing whether the measures from the simulation model are closely approximate to the measures expected from a proposed system (Law and McComas, 1991).

In this case, the exact measurement data are unavailable. Instead, this study addresses the unavailability of the data by using ABM. Therefore, the validation of this model is done based on the expected measure outcome. Particularly, this study uses the expected measurement discussed by Gregorioa and Ancog (2020), which approximated a 3.28% reduction of agricultural production in Indonesia. The reduced agricultural production is occurred due to the restriction caused by COVID-19, which leads to the reduced agricultural labour force (Vos et al., 2020).

The Goodness of Fit (GoF) is used to validate the model. GoF examines the observed data with the expected data using some measurement (Kéry and Royle, 2016; Stephenson, 2016). Particularly, the chi-square test is used to conduct the model validation. It decides whether the observed data from the simulation are “fit” or “close” enough to the expected data by assessing the null hypothesis $\left(H_{0}\right)$ and alternative hypothesis $\left(H_{a}\right)$ as given as follows:

(1) $$H_{0}:\text{There is no significant difference between the observed}\;\;\text{and expected data},H_{a}:\text{There is a significant difference between the observed}\;\;\text{and expected data}.$$

The null hypothesis cannot be rejected if and only if the chi-square value, denoted as $\chi^{2}$, is less than the critical value, vice versa. The formulation to calculate the $\chi^{2}$ is given as:

(2) $$\chi^{2}=\sum_{i=1}^{k}\frac{{\left(O_{i}-E_{i}\right)}^{2}}{E_{i}},$$

where $O_{i}$ represents the observed data, $E_{i}$ represents the expected data, and *k* represents the number of simulations run. In this case, the expected 3.28% reduction of agricultural production given by Gregorioa and Ancog (2020) is used as the expected data. Meanwhile, the rice production data obtained from the simulation result is used to calculate the difference production (percentage), either decreasing or increasing, between the production before and after COVID-19 emerged. The rice production during COVID-19 is said to be decreased (compared to the normal condition without COVID-19) if and only if the difference production percentage is positive, vice versa. A few samples of the observed rice production difference data obtained from one hundred simulations are given in Table 4.

**Table 4:** A few samples of observed rice production difference data from 100 simulations.

*i*-th simulation	Observed data (*O*_*i*_)	Expected data (*E*_*i*_)
1	0.061185	0.0328
2	0.059888	0.0328
3	0.059581	0.0328
4	0.045315	0.0328
5	0.032956	0.0328
⋮	⋮	⋮
96	-0.05063	0.0328
97	-0.04888	0.0328
98	-0.03877	0.0328
99	-0.03907	0.0328
100	-0.05932	0.0328

With a 5% level of significance, the critical value for 99 degrees of freedom is 123.225. The $\chi^{2}$ obtained is 11.934. Since the $\chi^{2}$ is less than the critical value, then the null hypothesis cannot be rejected. In other words, there is no significant difference between the observed and expected data.

### 3.2 Uncertain Multi-Objective Many-to-Many Location-Routing Problem

The uncertain model of Multi-Objective Many-to-Many Location-Routing Problem (MOMMLRP) used in our framework is based on the model proposed by Perdana et al. (2020) since this study is the development from the previous study to fill its research gap. The model is using two objectives: (1) maximize demand fulfilment and (2) minimize total operational cost. The operational cost consists of food hubs development cost, handling cost for rice, and distribution cost. Sets, parameters, and decision variables used in the uncertain model of MOMMLRP is given in Table 5, Table 6, and Table 7, respectively, where $f_{ck},d_{ci},b_{ji}\in Z$ and Z is a primitive uncertainty set. Primitive uncertainty set is an uncertainty set which has not been determined yet. In the previous work, box uncertainty set is used to gather all of the uncertainties. In this study, we used polyhedral uncertainty set to cover all of the uncertainties. The uncertain model of MOMMLRP which proposed by Perdana et al. (2020) is given as:

(3) $$\max\left\{\sum_{c\in C}\sum_{i\in I}v_{ci}\sum_{j\in J}w_{cji}\right\},$$

(4) $$\min\{h\sum_{j\in J}x_{j}+q\sum_{c\in C}\sum_{j\in J}P_{cj}+\sum_{c\in C}\sum_{j\in J}\sum_{i\in I}b_{ji}+d_{ci}w_{cji}\;\;+\sum_{c\in C}\sum_{k\in K}\sum_{j\in J}b_{kj}f_{ck}y_{ckj}\},$$

(5) $$\sum_{k\in K}f_{ck}y_{ckj}=P_{cj},\forall c\in C,j\in J,$$

(6) $$\sum_{i\in I}d_{ci}w_{cji}=P_{cj},\forall c\in C,j\in J,$$

(7) $$\sum_{j\in J}y_{ckj}\leq 1,\forall c\in C,k\in K,$$

(8) $$\sum_{j\in J}w_{cji}\leq 1,\forall c\in C,i\in I,$$

(9) $$y_{ckj}\leq x_{j},\forall c\in C,k\in K,j\in J,$$

(10) $$w_{cji}\leq x_{j},\forall c\in C,j\in J,i\in I.$$

**Table 5:** Sets used in the model.

Set	Description
*I*	Demand area
*J*	Potential food hub location
*K*	Production area
*C*	Food commodity

**Table 6:** Parameters used in the model.

Parameter	Description
*d*_*ci*_	Demand of commodity *c* in area *i* (ton/day)
*v*_*ci*_	Selling price of commodity *c* in area *i* (Rp/ton)
*f*_*ck*_	Production capacity of commodity *c* in area *k* (ton/day)
*b*_*ji*_	Distribution cost between area *j* and *i* (Rp/ton)
*q*_*c*_	Handling cost for commodity *c* (Rp/ton)
*h*	Food hubs development cost (Rp/unit)

**Table 7:** Decision variables used in the model.

Decision variables	Description
*x*_*j*_ ∈ {0,1}	Is food hub will developed in area *j*?
$P_{cj}\in R$	Food hub capacity of commodity *c* in area *j* (ton/day)
*y*_*ckj*_ ∈ [0,1]	Food distribution ratio of commodity *c* distributed from production area *k* to food hub in area *j* (proportional to food production capacity *f*_*ck*_)
*w*_*cji*_ ∈ [0,1]	Food distribution ratio of commodity *c* distributed from food hub in area *j* to fulfill the demand in area *i* (proportional to food demand *d*_*ci*_)

The objective function (3) is maximizing the demand fulfilment. Meanwhile, the objective function (4) is minimizing the total operational cost needed. Constraint function (5) guarantees that each commodity's capacity in each developed food hub is adjusted based on the total food distributed from all food production zone. Constraint (6) guarantees that each commodity's capacity in each developed food hub is adjusted based on the total food distributed to all demand areas. Constraint (5) and (6) describe the distribution flow equation. Constraint (7) guarantees that all food distributed from each production zone does not exceed its food production capacity. Constraint (8) guarantees that all of the food distributed from developed food hub to each demand area does not exceed its food demand. Constraint (9) and (10) guarantee that no food will be distributed to and from the potential food hub if it is not developed. In other words, food will be distributed to and from the potential food hub only if the potential food hub is developed.

### 3.3 Framework integration of Agent-Based Modelling and Robust Optimization

**Figure 3:**
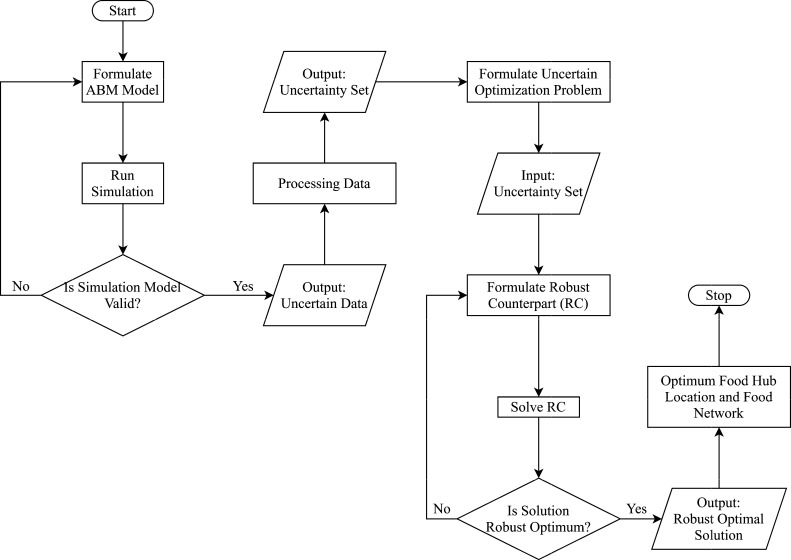
Framework of integrated ABM and RO.

This study integrates ABM and RO to design the robust optimum rice supply chain involving normal and pandemic conditions. ABM is used to simulate the rice production activity during normal and pandemic conditions to produce rice production capacity output. The rice production capacity data from ABM simulation are then used in RO under two scenarios: (1) using all the production capacity data and (2) using the highest 15% of rice production capacity data. Then, each production capacity dataset in both scenarios is processed to build the polyhedral uncertainty set. After the polyhedral uncertainty set is generated, we can construct the RMOMMLRP and solve the model to obtain the robust optimum rice supply chain strategy. The framework of the integrated ABM and RO is given in Fig. 3.

This framework is started with the formulation of the ABM simulation model. Then, the formulated ABM model is run and tested whether it is valid or not. The formulation of the ABM simulation model is repeated until the valid simulation model is obtained. Once the simulation model is valid, the output data will be processed to construct the two scenarios. Afterwards, data from the two scenarios are further processed to acquire the uncertainty set, which will be used in RO. RO transforms the uncertain optimization problem into RC with respect to the uncertainty set acquired from the previous step. Once the RC is obtained, it is solved and checked whether the solution is robust optimum or not. The RC formulation is also repeated until the robust optimal solution is obtained. Afterwards, the robust optimal solution is interpreted to give the optimum strategy of the rice supply chain involving the COVID-19 pandemic.

## 4 Results and discussion

In this paper, we developed Robust MOMMLRP (RMOMMLRP) model to handle the uncertainties in the uncertain MOMMLRP as discussed in Subsection 3.2 Notably, the uncertainties are considered to exist in the polyhedral uncertainty set. Furthermore, a small case study is given as an example of how ABM and RO are integrated. We took five regions with the most positive cases of COVID-19 in West Java, i.e., Depok City, Bogor City, Bogor Regency, Bekasi City, and Bekasi Regency. Netlogo 6.1.0. is used to run the simulation and import the rice production capacity results in each region. Then, we solve the RMOMMLRP using RStudio and obtain the robust optimum food hub location with the rice supply network.

### 4.1 Robust Multi-Objective Many-to-Many Location-Routing Problem using polyhedral uncertainty set

In this study, we consider the uncertain parameters exist in polyhedral uncertainty set, which defined as:

(11) $$U_{poly}=\left\{\zeta_{i}:-M_{i}\zeta_{i}+q_{i}\geq 0\right\}$$

where $\zeta_{i}$ is the uncertain parameter. By following several assumptions of robust optimization as discussed by Ben-Tal et al. (2009) and Yanıkoğlu et al. (2019), one can formulate the RC of uncertain MOMMLRP, or also known as Robust Multi-Objective Many-to-Many Location-Routing Problem (RMOMMLRP), as given below:

(12) $$\max\left\{\sum_{c\in C}\sum_{i\in I}v_{ci}\sum_{j\in J}w_{cji}\right\},$$

(13) $$\min\left\{z+h\sum_{j\in J}x_{j}+q\sum_{c\in C}\sum_{j\in J}P_{cj}\right\},$$

(14) $$\sum_{k\in K}f_{ck}y_{ckj}+\sum_{g=1}^{H_{3}}{\left(q_{3}\right)}_{g}{\left(v_{3}\right)}_{g}=P_{cj},\forall c\in C,j\in J,$$

(15) $$\sum_{g=1}^{H_{3}}{\left(v_{3}\right)}_{g}{\left(M_{3}\right)}_{gl}=\sum_{k\in K}y_{ckj}{\left(D_{3}\right)}_{ckjl},\forall c\in C,j\in J,l=1,2,\cdots ,L_{3},$$

(16) $${\left(v_{3}\right)}_{g}\geq 0,\forall g=1,2,\cdots ,H_{3},$$

(17) $$\sum_{i\in I}d_{ci}w_{cji}+\sum_{g=1}^{H_{4}}{\left(q_{4}\right)}_{g}{\left(v_{4}\right)}_{g}=P_{cj},\forall c\in C,j\in J,$$

(18) $$\sum_{g=1}^{H_{4}}{\left(v_{4}\right)}_{g}{\left(M_{4}\right)}_{gl}=\sum_{i\in I}w_{cji}{\left(D_{4}\right)}_{cjil},\forall c\in C,j\in J,l=1,2,\cdots ,L_{4},$$

(19) $${\left(v_{4}\right)}_{g}\geq 0,\forall g=1,2,\cdots ,H_{4}$$

(20) $$\sum_{j\in J}y_{ckj}\leq 1,\forall c\in C,k\in K,$$

(21) $$\sum_{j\in J}w_{cji}\leq 1,\forall c\in C,i\in I,$$

(22) $$y_{ckj}\leq x_{j},\forall c\in C,k\in K,j\in J,$$

(23) $$w_{cji}\leq x_{j},\forall c\in C,j\in J,i\in I,$$

(24) $$\sum_{c\in C}\sum_{j\in J}\sum_{i\in I}m_{cji}w_{cji}+\sum_{c\in C}\sum_{k\in K}\sum_{j\in J}n_{ckj}y_{ckj}+\sum_{g=1}^{H_{1}}{\left(q_{1}\right)}_{g}{\left(v_{1}\right)}_{g}\;\;+\sum_{g=1}^{H_{2}}{\left(q_{2}\right)}_{g}{\left(v_{2}\right)}_{g}\leq z,$$

(25) $$\sum_{g=1}^{H_{1}}{\left(v_{1}\right)}_{g}{\left(M_{1}\right)}_{gl}=\sum_{c\in C}\sum_{j\in J}\sum_{i\in I}w_{cji}{\left(D_{1}\right)}_{cjil},\forall l=1,2,\cdots ,L_{1}$$

(26) $$\sum_{g=1}^{H_{2}}{\left(v_{2}\right)}_{g}{\left(M_{2}\right)}_{gl}=\sum_{c\in C}\sum_{j\in J}\sum_{i\in I}y_{ckj}{\left(D_{2}\right)}_{ckjl},\forall l=1,2,\cdots ,L_{2}$$

(27) $${\left(v_{1}\right)}_{g}\geq 0,\forall g=1,2,\cdots ,H_{1},$$

(28) $${\left(v_{2}\right)}_{g}\geq 0,\forall g=1,2,\cdots ,H_{2},$$

### 4.2 Agent-Based Modelling simulation result

**Figure 4:**
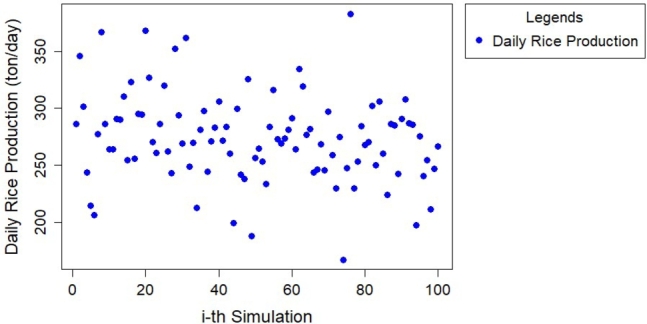
Daily rice production in Bekasi Regency for scenario 1.

As stated by Pérez-Salazar et al. (2019), one of the main challenges in the agri-food supply chain is the uncertain variations of quantity in primary production. Therefore, in this study, an ABM is developed to simulate the rice production activity in rice milling units and give the rice production capacity during the normal and COVID-19 pandemic. This type of ABM simulation is classified as a realistic model since it involves realistic mechanisms (Utomo et al., 2020). The simulation begins with the normal condition for the first year. Afterwards, the COVID-19 condition is set to emerge in the simulation for the following year. From the ABM simulation, we obtained daily data of rice production capacity for each region. Then, the data are processed to construct two scenarios: (1) use all the production capacity data retrieved from simulation and (2) use the highest 15% of rice production capacity data only. For example, the whole daily rice production capacity data in Bekasi Regency is given in Fig. 4, which is referred to as scenario 1. Meanwhile, the highest 15% of daily rice production capacity data in Bekasi Regency is presented in 5, which is referred to as scenario 2.

**Figure 5:**
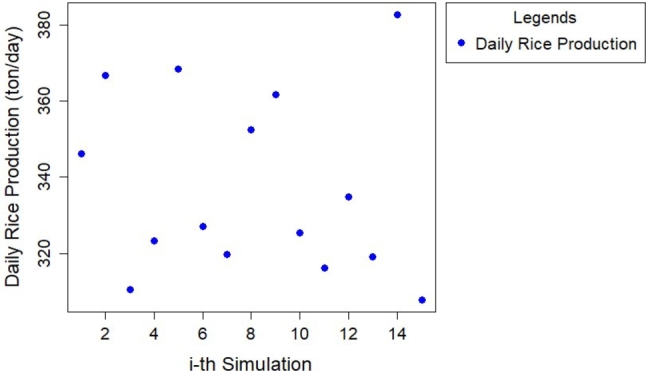
Daily rice production in Bekasi Regency for scenario 2.

Then, the rice production capacity in each region is used in RO to generate the polyhedral uncertainty set for both scenarios, which covers the uncertainties. In other words, there will be two polyhedral uncertainty sets for each region, which represents the data from the two scenarios.

### 4.3 Robust Optimization result

**Figure 6:**
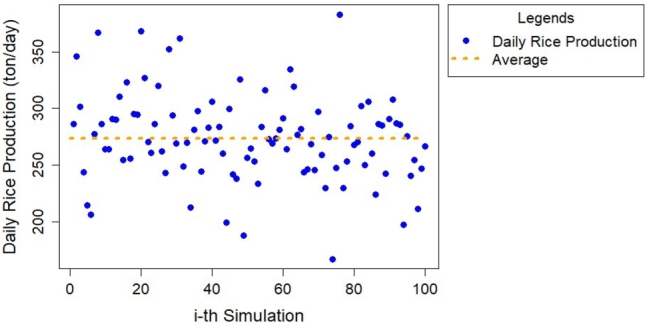
Daily average of rice production in Bekasi Regency for scenario 1.

As discussed in Subsection 4.2, we generate the polyhedral uncertainty set based on the data produced from the simulation. In this study, we use the daily average production value to be the nominal data. As an example, the daily average production value in Bekasi Regency for scenario 1 is given in Fig. 6.

**Figure 7:**
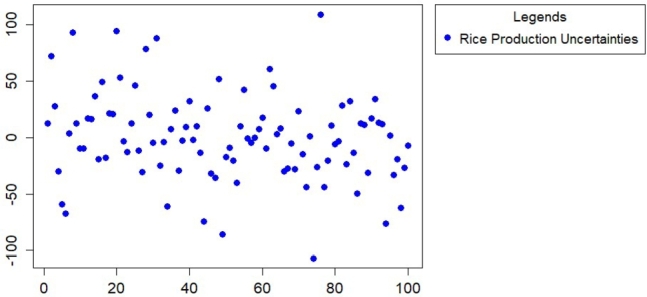
Uncertainties of daily rice production in Bekasi Regency for scenario 1.

The uncertainties are the difference between the nominal data, which is the average value, and its daily rice production. In other words, the uncertainties are the deviation of rice production from its average value. Thus, we obtained the uncertainties of daily rice production in Bekasi Regency for scenario 1 as given in Fig. 7.

After this, we can build the polyhedral uncertainty set for the uncertain rice production capacity in Bekasi Regency in scenario 1. By recalling the definition of the polyhedral uncertainty set, we should construct the inequality system to gather all of the uncertain data. For the uncertain rice production capacity in Bekasi Regency in scenario 1, the inequality system is given in the inequality system (29).

(29) $$u_{3}\leq 59.8879217\cdot t-47.22553\;u_{3}\leq 3.4066680\cdot t+65.73698\;u_{3}\leq 0.2374074\cdot t+91.09106\;u_{3}\leq -4.8441951\cdot t+477.29285\;u_{3}\geq -17.9989243\cdot t+30.66131\;u_{3}\geq -8.3322326\cdot t-17.67214\;u_{3}\geq -0.5743727\cdot t-64.21930\;u_{3}\geq 1.5297458\cdot t-219.92407\;u_{3}\geq 3.5151606\cdot t-406.55306\;u_{3}\geq 27.4703293\cdot t-2754.15959$$

**Figure 8:**
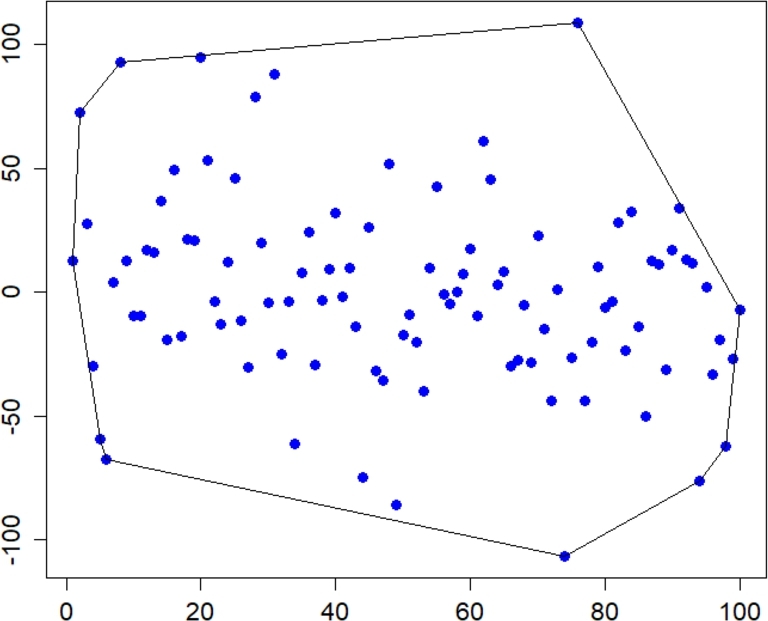
Uncertainty set of daily rice production in Bekasi Regency for scenario 1.

By defining the Matrix **M** and vectors ζ,q, we have defined the polyhedral uncertainty set for the uncertain rice production capacity in Bekasi Regency. The polyhedral uncertainty set obtained for rice production capacity in Bekasi Regency in scenario 1 is illustrated in Fig. 8. Note that this is also applied to other regions.

**Figure 9:**
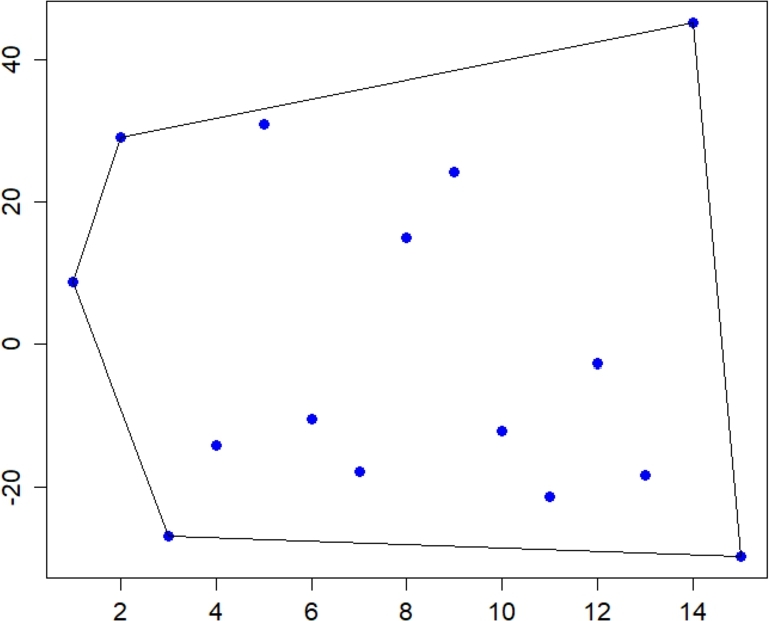
Uncertainty set of daily rice production in Bekasi Regency for scenario 2.

By doing the same steps, one can obtain the polyhedral uncertainty set for Bekasi Regency in scenario 2 as given by the inequality system (30) and illustrated in Fig. 9. Note that the RMOMMLRP will be solved for both of the scenarios independently based on their respective polyhedral uncertainty set. Hereinafter, we can solve the RMOMMLRP using the obtained polyhedral uncertainty set to gather the robust optimum rice supply chain strategy for the two scenarios.

(30) $$u_{3}\leq 20.4400081\cdot t-11.68248\;u_{3}\leq 1.3453084\cdot t+26.50691\;u_{3}\leq -75.1202844\cdot t+1097.02521\;u_{3}\geq -17.8361854\cdot t+26.59371\;u_{3}\geq -0.2386837\cdot t-26.19880$$

### 4.4 Robust optimum rice supply chain strategy

Solving the RMOMMLRP provides robust optimum food hub locations for scenario 1 as given in Table 8. Since scenario 1 uses all rice production data retrieved from ABM simulation, RO seeks the best result given the worst case possible from all of the data.

**Table 8:** Robust optimum food hub location in scenario 1.

Food hub location	Capacity (ton)
Depok City	826.323

The robust optimum rice supply from producer area to food hub in scenario 1 is presented in Table 9. It shows that Depok City is not produced any rice based on the worst-scenario. It also shows that all of the rice produced in every region are distributed to maximize the demand fulfillment.

**Table 9:** Robust optimum rice supply from producer area to food hub in scenario 1.

Producer area	Production capacity (ton)	Supplied to food hub	Quantity supplied (ton)	Supplied percentage (%)
Bekasi City	0,911	Depok City	0,911	100%
Bogor City	2,734	Depok City	2,734	100%
Depok City	-	-	-	-
Bekasi Regency	166,905	Depok City	166,905	100%
Bogor Regency	655,773	Depok City	655,773	100%

Even though the whole rice produced have been distributed, the overall demand in scenario 1 could not be completely fulfilled as shown in Table 10. In other words, the rice produced in all five regions is insufficient to fulfil their overall demand in scenario 1. Thus, the whole available rice is prioritized to be distributed to satisfy the regions which have the largest selling price in order to balance the market price and maximize the welfare of local farmers as well as strengthening the local food supply chain (Kharisma and Perdana, 2019; Bashiri et al., 2021; Béné, 2020).

**Table 10:** Robust optimum demand fulfillment in scenario 1.

Food hub location	Service area	Demand (Ton)	Demand fulfilled	
			Ton	%
Depok City	Depok City	628,294	628,294	100,00%
	Bekasi City	790,486	198,029	25,05%

**Figure 10:**
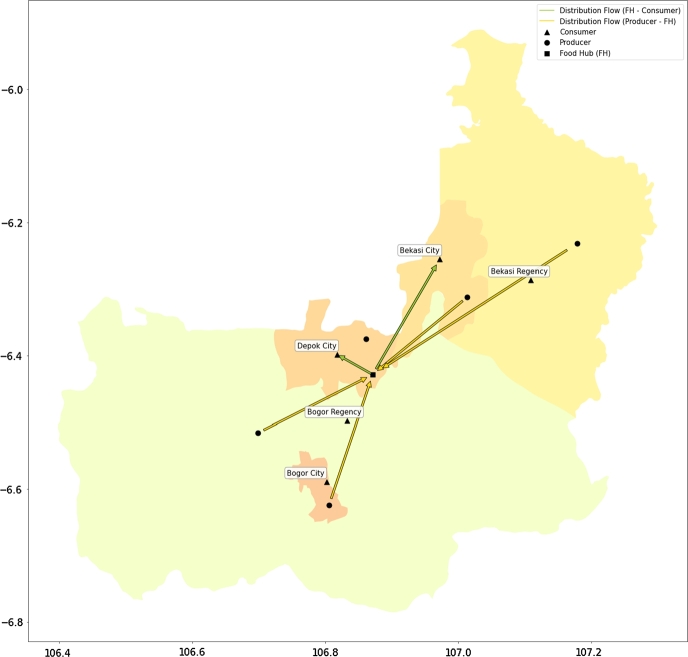
Robust optimum rice supply network in scenario 1.

In this case, Depok City has the highest rice selling price. The rice selling price is observed between June 2020 - June 2021 from The National Strategic Food Price Information Center (PIHPS), managed by Bank Indonesia. Since Depok City has the highest selling price, all demand in Depok City is prioritized to be fulfilled. The second highest selling price is in the Bekasi City and Bekasi Regency. However, Bekasi City is chosen to be the service area of the food hub in Depok City since it is closer than Bekasi Regency. Hence, the remainder of available rice is focused on fulfilling the demand in Bekasi City. The complete rice supply network between the producer area, food hub location, and consumer base for scenario 1 is given in Fig. 10.

For scenario 2, the robust optimum food hub locations are given in Table 11. Since scenario 2 uses the highest 15% of rice production volumes rather than the whole data, the worst-case food production volume in scenario 2 is higher than scenario 1, which is less conservative and leads to higher demand fulfilment.

**Table 11:** Robust optimum food hub location in scenario 2.

Food hub location	Capacity (ton)
Depok City	1279,128
Bekasi Regency	382,892

The robust optimum rice supply from producer area to food hub in scenario 2 is presented in Table 12. At the highest 15% of rice production volume, Depok City is now producing their own rice. Similar to the scenario 1, all of the rice produced in every region are distributed to maximize the demand fulfillment.

**Table 12:** Robust optimum rice supply from producer area to food hub in scenario 2.

Producer area	Production capacity (ton)	Supplied to food hub	Quantity supplied (ton)	Supplied percentage (%)
Bekasi City	75,250	Bekasi Regency	75,250	100%
Bogor City	80,719	Depok City	80,719	100%
Depok City	64,705	Depok City	64,705	100%
Bekasi Regency	307,642	Bekasi Regency	307,642	100%
Bogor Regency	1133,704	Depok City	1133,704	100%

Even though the 15% highest rice production volume is used, the overall rice demand in the five regions is still could not be fulfilled entirely on their own as presented in Table 13. Rice produced in Bogor Regency, Bogor City and Depok City are supplied to the food hub in Depok City since it is the nearest food hub. Then, the rice collected by the food hub in Depok City is supplied to Depok City itself since it has the highest selling price among the five regions. The remaining rice in the food hub of Depok City will be sent to other regions with the second-highest selling price. In this case, Bekasi City and Bekasi Regency share the same selling price as the second highest. However, Bekasi City is chosen to be the next service area from the food hub in Depok City since it is closer than Bekasi Regency.

**Table 13:** Robust optimum demand fulfillment in scenario 2.

Food hub location	Service area	Demand (Ton)	Demand fulfilled	
			Ton	%
Depok City	Bekasi City	790,486	650,834	82,33%
	Depok City	628,294	628,294	100.00%
Bekasi Regency	Bekasi Regency	978,951	382,892	39,11%

**Figure 11:**
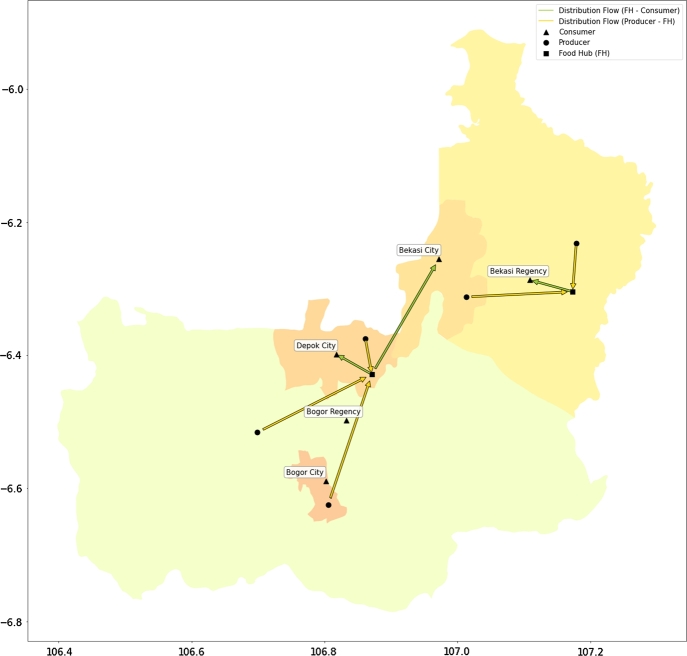
Robust optimum rice supply network in scenario 2.

Meanwhile, rice from Bekasi City and Bekasi regency is sent to the food hub in Bekasi Regency since it is the nearest food hub considering the unfulfilled demand in both regions with the second-highest selling price. Then, the rice collected by the food hub in Bekasi Regency is used to fulfil the demand in Bekasi Regency. The complete rice supply network for scenario 2 is given in Fig. 11.

To increase the demand fulfilment, it is needed to connect to other nearest possible producer areas with large production capacity. The closer it's connected to, the better it becomes. Apart from the connectivity issue, less distance among the food supply chains helps to sustain the better freshness of food and lower the risk of being perished (Pal and Kant, 2017; Jouzdani et al., 2020; Govindan et al., 2014). Less distance also leads to less energy used (Rossi et al., 2020; Grebitus et al., 2013). These factors are essential to maintain the sustainability of the food supply chain (Govindan et al., 2020; Sherafati et al., 2020).

Moreover, less distance among the food supply chains could also lower the risk of food being exposed to the virus (Béné, 2020). Food safety and hygiene should be ensured at each level of the food supply chain, from the producer down to the consumer (Rizou et al., 2020; Lacombe et al., 2020). The safety of the workers along the food supply chain should also be ensured (Waltenburg et al., 2021; Flocks, 2020).

Empowering the local food supply chain is vital in the meantime of COVID-19 since it is less vulnerable to connectivity disruptions (Hobbs, 2020). Moreover, a shorter food supply chain enables the producer to develop a direct relationship with their partners and customers, promoting the strength of the local food supply chain (Thilmany et al., 2021). The development of the local food supply chain has been proved to plays a significant role in the resiliency of the food network (Fei et al., 2020).

## 5 Conclusion

In many optimization problems, we often met several uncertainties. To handle the uncertain problems, RO is used by considering the uncertainties to be exist in an uncertainty set. Nevertheless, the actual uncertain data might be unavailable. This study gives a framework for integrating ABM simulation and RO to handle the actual data availability issues in rice supply chain problems involving COVID-19 and normal conditions. In this study, ABM is used to simulate the pandemic spread and its impact on rice production capacity. The simulation aims to give uncertain data of rice production capacity during normal and pandemic conditions. Then, the uncertain rice production capacity data are used in RO to define the uncertainty set in the problem. The robust optimum rice supply chains strategy is obtained based on the global robust optimal solution given by the RC of the uncertain problem. The RC of the uncertain problem is computationally tractable with respect to the uncertainty set.

The robust optimum rice supply chain strategy considering the normal and COVID-19 conditions is in line with the local food supply chain principle. The local food supply chain helps to ensure food security. It also ensures the sustainability issues, from the increasing welfare of the farmers to lowering the perished food and energy used as environmental problems. Furthermore, it helps lower the risk of food being exposed to the virus and maintain food safety.

There are several gaps in this study. One of the gaps is based on the food supply chain actors covered by the simulation. In this study, we give an example of how to simulate the upstream actor of the food supply chain, particularly the rice milling workers. It is also interesting to simulate the consumption behaviour of downstream actors in the food supply chain given the stimulus of a particular condition, or in this case, is the impact of the COVID-19 pandemic on food consumption behaviour. Another gap is based on the simulation methods used. ABM is useful to simulate a system in which the components/actors/agents are easily identified along with their behaviour and other attributes. In this case, ABM is suited to the problem of the food production system since it is easy to identify the components/actors/agents of the upstream actor in the food supply chain. It is important to note that the methods of simulation used may vary, depending on the problem addressed. Thus, one can develop this framework and adjust the simulation methods used to be integrated with RO.

## Declarations

### Author contribution statement

Tomy Perdana: Conceived and designed the experiments; Performed the experiments; Analyzed and interpreted the data; Wrote the paper.

Audi Luqmanul Hakim Achmad and Diah Chaerani: Conceived and designed the experiments; Performed the experiments; Contributed reagents, materials, analysis tools or data; Wrote the paper.

### Funding statement

This work was supported by the Indonesian 10.13039/501100020638Ministry of Research and Technology/National Research and Innovation Agency through contract number 1207/UN6.3.1/PT.00/2021 entitled “Coordination Model of Food Supply Chain Management in COVID-19 Pandemic”.

### Data availability statement

Data included in article/supplementary material/referenced in article.

### Declaration of interests statement

The authors declare no conflict of interest.

### Additional information

No additional information is available for this paper.
